# Involvement of GSK-3β Phosphorylation Through PI3-K/Akt in Cerebral Ischemia-Induced Neurogenesis in Rats

**DOI:** 10.1007/s12035-016-0290-8

**Published:** 2016-11-19

**Authors:** Keishi Kisoh, Hideki Hayashi, Tsuyoshi Itoh, Mayumi Asada, Miho Arai, Bo Yuan, Kouichi Tanonaka, Norio Takagi

**Affiliations:** 1 Department of Applied Biochemistry, Tokyo University of Pharmacy and Life Sciences, 1432-1 Horinouchi, Hachioji, Tokyo 192-0392 Japan; 2 Department of Molecular and Cellular Pharmacology, Tokyo University of Pharmacy and Life Sciences, 1432-1 Horinouchi, Hachioji, Tokyo 192-0392 Japan

**Keywords:** Cerebral ischemia, Neurogenesis, GSK-3β, PI3-K/Akt

## Abstract

Glycogen synthase kinase (GSK)-3β, which is abundantly expressed in the central nervous system, regulates various cellular processes including gene expression, cell proliferation, and differentiation. However, involvement of GSK-3β in cerebral ischemia-induced endogenous neurogenesis is not yet fully understood. Appropriate strategies to prevent ischemic cell damage and subsequent severe sequelae are needed. The purpose of the present study was to determine the relationship between pathophysiological alteration of the GSK-3β signaling pathway and cerebral ischemia-induced endogenous neurogenesis in rats. Severe cerebral ischemia was produced by the injection of 700 microspheres into the right internal carotid artery of rats. We demonstrated that phosphorylation of GSK-3β at its Ser9 and that of Akt was significantly enhanced on day 7 after the cerebral ischemia, as was the number of NeuroD-positive cells. Treatment with a phosphatidylinositol 3-kinase (PI3-K) inhibitor decreased the cerebral ischemia-induced phosphorylation of Akt and that of GSK-3β at its Ser9. In addition, as the protein levels of insulin-like growth factor-1 (IGF-1) and brain-derived neurotrophic factor (BDNF) were decreased, they might not have been essential for activation of the PI3-K/Akt/GSK-3β pathway after severe cerebral ischemia. Although it remains to be determined what factors activate this pathway, our results suggest that PI3K/Akt-dependent GSK-3β signaling and subsequent expression of NeuroD were involved in the neurogenesis elicited by cerebral ischemia.

## Introduction

Glycogen synthase kinase-3 (GSK-3), a serine/threonine (Ser/Thr) kinase, was identified some time ago as an enzyme that phosphorylates glycogen synthase to render it inactive [1]. There are two isoforms of it, i.e., α and β. GSK-3β is abundantly expressed in the mammalian central nervous system and regulates various cellular processes including gene expression, cell proliferation, and differentiation. β-Catenin is a signaling molecule that is phosphorylated by GSK-3β, resulting in its degradation by the ubiquitin-proteasome system. Thus, inactivated GSK-3β, which is phosphorylated at its Ser9, inhibits this proteasomal degradation of β-catenin. As a result, β-catenin accumulates in the cytosol and is translocated into the nucleus, where it contributes to gene expression related to cell proliferation and differentiation [2, 3]. Therefore, β-catenin has been implicated in the maintenance of the stem cell pool [4], neuronal differentiation [5], and the development of the central nervous system [6]. NeuroD, which is a proneural basic helix-loop-helix (bHLH) transcription factor, promotes premature cell cycle exit and differentiation into neural progenitor cells, indicating a differentiation factor [7, 8]. Its expression is regulated by β-catenin [9]. Some studies have indicated that NeuroD is also involved in not only embryonic neurogenesis but also in postnatal neurogenesis [10, 11]. Furthermore, environmental signals regulate adult neurogenesis, at least in part, through the activation of NeuroD [12, 13].

It has been shown that neurogenesis occurs in two restricted areas of the brain, i.e., the subventricular zone of the lateral ventricles and subgranular zone (SGZ) of hippocampal dentate gyrus, throughout life [14]. Furthermore, the existence of additional stem cell niches for neurogenesis was demonstrated, such as the substantia nigra, circumventricular organs located along the ventricular midline, and the walls of the third and fourth ventricles [15–17]. The process of neurogenesis is closely related to neurological functions involving spatial learning, memory, and behavioral response [18] and is regulated by various factors such as age, drugs, disease, and the environment. Indeed, anti-depressants improve depression symptoms, and there is a correlation between their use and restoration of impaired neurogenesis in the hippocampus [19]. Cerebral ischemia also enhances neurogenesis [20–22]. In addition, it has been implied that neurons newly generated after cerebral ischemia migrate to the injured area [23, 24] and replace the injured or dead neurons [20]. Some of these cells might be integrated into the existing neural network or form new ones. Therefore, acceleration of endogenous neurogenesis in the brain has been expected as new therapeutic approach for cerebral ischemia. However, it has been shown that the majority of newly generated endogenous neurons fail to survive. Rather, neural stem/progenitor cells without proliferation activity secrete autocrine and/or paracrine factors to maintain the multipotency in the central nervous system [25]. Furthermore, neural stem cells, when transplanted into lesion sites in an injured spinal cord, have the ability to increase the expression of neurotrophic factors [26, 27]. In this sense, we demonstrated previously that the injection of neural progenitor cells (NPCs), which were isolated by using the neurosphere method, results in improvement of spatial learning dysfunction and post-stroke depression [28, 29]. However, the pathophysiological alterations leading to cerebral ischemia-induced endogenous neurogenesis are not fully understood. Therefore, it is important to investigate the pathophysiological environment underlying cerebral ischemia-induced neurogenesis for appropriate therapies for stroke. Elucidating the regulation of endogenous neurogenesis in cerebral ischemia may lead to a further understanding of the pathophysiology of stroke and the development of new therapeutic targets. The aim of this study was to determine the relationship between pathophysiological alteration of the GSK-3β signaling pathway and the initial stages of cerebral ischemia-induced endogenous neurogenesis in rats.

## Materials and Methods

### Animal Surgical Procedures

Male Wistar rats weighing 220–250 g (Charles River Japan Inc., Tsukuba, Japan) were used. The rats were maintained at 23 ± 1 °C in a room with a constant humidity of 55 ± 5% and a light cycle of 12-h light/12-h darkness. The rats had free access to food and water according to the National Institute of Health Guide for the Care and Use of Laboratory Animals and the Guidance for Experimental Animal Care issued by the Prime Minister’s Office of Japan. The study was approved by the Committee of Animal Care and Welfare of Tokyo University of Pharmacy and Life Sciences. Microsphere-induced cerebral embolism (ME) was performed by the method described previously [30]. Anesthesia was induced with 4% isoflurane and maintained with 2.5% isoflurane. The right external carotid and pterygopalatine arteries were temporarily occluded with strings. Immediately thereafter, a needle connected to a polyethylene catheter (TORAY Feeding Tube, Chiba, Japan) was inserted into the right common carotid artery. Next, 700 microspheres (45.0 μm in diameter; Polysciences Inc., Warrington, PA, USA), suspended in 20% dextran solution (150 μl), were injected over a 20-s period into the right internal carotid artery through the cannula. After the injection, the needle was removed, and the puncture wound was then repaired with surgical glue. Sham-operated rats received the same volume of vehicle without microspheres. Non-operated rats were used as naïve control rats in the present study. As described in earlier reports [31, 32], scattered necrotic areas, variable in size and shape, were seen mainly in the parietotemporal cortex, corpus callosum, hippocampus, thalamus, and lenticular nucleus of the ipsilateral hemisphere after microsphere embolism. In the present study, 41 ME rats were prepared for experiments. Among these rats, 6 of them (14.6%) died within 3 days after the operation. Twenty-five sham-operated rats survived throughout the experiments.

Wortmannin (Sigma-Aldrich, Inc. MO, USA), which is a potent and selective inhibitor of phosphatidylinositol 3-kinase (PI3-K; IC_50_ = 2 nM; [33]), was dissolved in dimethyl sulfoxide and injected intravenously, as previously reported [34, 35], on days 5 and 6 after ME. For tissue sampling for Western blotting and histochemical analysis, wortmannin-untreated and wortmannin-treated cerebral ischemic rats were sacrificed on day 7 after surgery. The dose used in the present study was based on the data obtained in our preliminary study: the effect of treatment with 30 μg/kg wortmannin on phosphorylation of Akt was more stable than that with 15 or 100 μg/kg.

### Western Immunoblotting

Rats were sacrificed by decapitation on days 1, 3, 7, and 14 day after surgery for cerebral ischemia- or sham-operated rats (*n* = 5 each). To determine the effects of wortmannin on the phosphorylation of Akt, wortmannin-treated cerebral ischemic rats were sacrificed by decapitation on day 7 after surgery (*n* = 5). The ipsilateral hemisphere was homogenized in ice-cold buffer containing 320 mM sucrose, protease inhibitors, and phosphatase inhibitor cocktail (Roche Diagnostics Co.) at 4 °C. The homogenate was centrifuged at 1000*g* for 10 min at 4 °C. The supernatant was then collected and subsequently centrifuged at 12,000*g* for 20 min. After this second centrifugation, the supernatant was collected and centrifuged at 100,000*g* in a TLA120.2 rotor (Beckman Coulter) for 60 min at 4 °C. After centrifugation, the supernatant was collected and used as the cytosolic fraction. Western blotting using total homogenate and cytosolic fraction was performed according to standard protocols. The following primary antibodies were used: rabbit monoclonal anti-phospho-GSK-3β (Ser 9; Cat. No. 5558, Cell Signaling Technology Inc., Danvers, MA, USA), mouse monoclonal anti-phospho-GSK-3α/β (Tyr 279/Tyr 216; Cat. No. 05-413, Upstate Biotechnology Inc.), rabbit monoclonal anti-GSK-3β (Cell Signaling), rabbit monoclonal anti-phospho-Akt (Cell Signaling), rabbit monoclonal anti-Akt (Cat. No. 4691, Cell Signaling), rabbit polyclonal anti-phospho-β-catenin (Cat. No. 9561, Cell Signaling), mouse monoclonal anti-β-catenin (Cat. No. ab6301; Abcam, Minneapolis, MN, USA), goat polyclonal anti-NeuroD (Cat. No. SC-1084, Santa Cruz), mouse monoclonal anti-GAPDH (Cat. No. MAB374, Millipore, Massachusetts, USA), rabbit polyclonal anti- brain-derived neurotrophic factor (BDNF; Cat. No. SC-546, Santa Cruz), rabbit polyclonal anti- insulin-like growth factor-1 (IGF-1; Cat. No. SC-9013, Santa Cruz), and mouse monoclonal anti-β-actin (Cat. No. A1978, Sigma-Aldrich) antibody. Subsequently, the protein blots were washed and incubated with the appropriate secondary antibodies. Bound antibodies were detected by the enhanced chemiluminescence method (Amersham Biosciences Inc., Piscataway, NJ, USA). Quantification was performed by using computerized densitometry (LuminographII, ATTO Co., Tokyo, Japan) and an image analyzer (CS Analyzer, ATTO Co., Tokyo, Japan).

### Immunohistochemistry

On day 7 after surgery, wortmannin-untreated (*n* = 5) and wortmannin-treated (*n* = 5) cerebral ischemic rats were perfused via the heart with 4% paraformaldehyde in 0.1 M phosphate buffer. The brains were quickly removed and immersed in 30% sucrose in 0.1 M phosphate buffer. They were then cut into 5-mm-thick coronal slabs, which were subsequently embedded in Neg50 (Richard-Allan Scientific, Kalamazoo, MI, USA) and cut into 20-μm sections with a cryostat. For immunostaining, sections were incubated overnight with the desired primary antibody at 4 °C after blocking, and then with the corresponding secondary antibody for 1 h at 25 °C. In the case of double immunofluorescence staining, after a wash, the same section was incubated overnight with another primary antibody at 4 °C. Subsequently, it was incubated with the corresponding secondary antibody for 1 h at 25 °C. Omission of primary antibodies served as a negative control. No immunostaining was detected in this group. The following primary antibodies were used: mouse monoclonal anti-Ki67 (Cat. No. 556003, BD Bioscience, San Jose, USA), goat polyclonal anti-doublecortin (DCX) (Cat. No. SC-8066, Santa Cruz Biotechnology Inc., Santa Cruz, CA, USA), or goat polyclonal anti-NeuroD (Cat. No. SC-1084, Santa Cruz) antibody. The secondary antibodies used were as follows: Alexa Fluor 488-labeled donkey anti-mouse IgG (Molecular Probes Inc., Eugene, OR, USA) and Alexa Fluor 594-labeled donkey anti-goat IgG antibodies (Molecular Probes Inc.). Fluorescence was detected by using an Olympus fluorescence microscope (IX-71; Olympus). Fluorescent images were loaded into the MetaMorph software program (Molecular Devices, Downingtown, PA). Based on background fluorescence and the size of their nucleus, NeuroD-labeled cells in the SGZ and granule cell layer (GCL) of the hippocampal dentate gyrus were counted by use of the MetaMorph software program in five sections per animal, which areas corresponded to coronal coordinates of −3.14 to −4.52 from bregma.

### Statistical Analysis

The results were expressed as the means ± standard error of the mean (SEM). Differences between two groups were evaluated statistically by use of the unpaired Student’s *t* test. Statistical comparison among multiple groups was evaluated by analysis of variance (ANOVA), followed by Bonferroni test as a post hoc test. *P* values of less than 0.05 were considered significant.

## Results

### GSK-3β/β-Catenin Signaling Pathway After ME

**Fig. 1:**
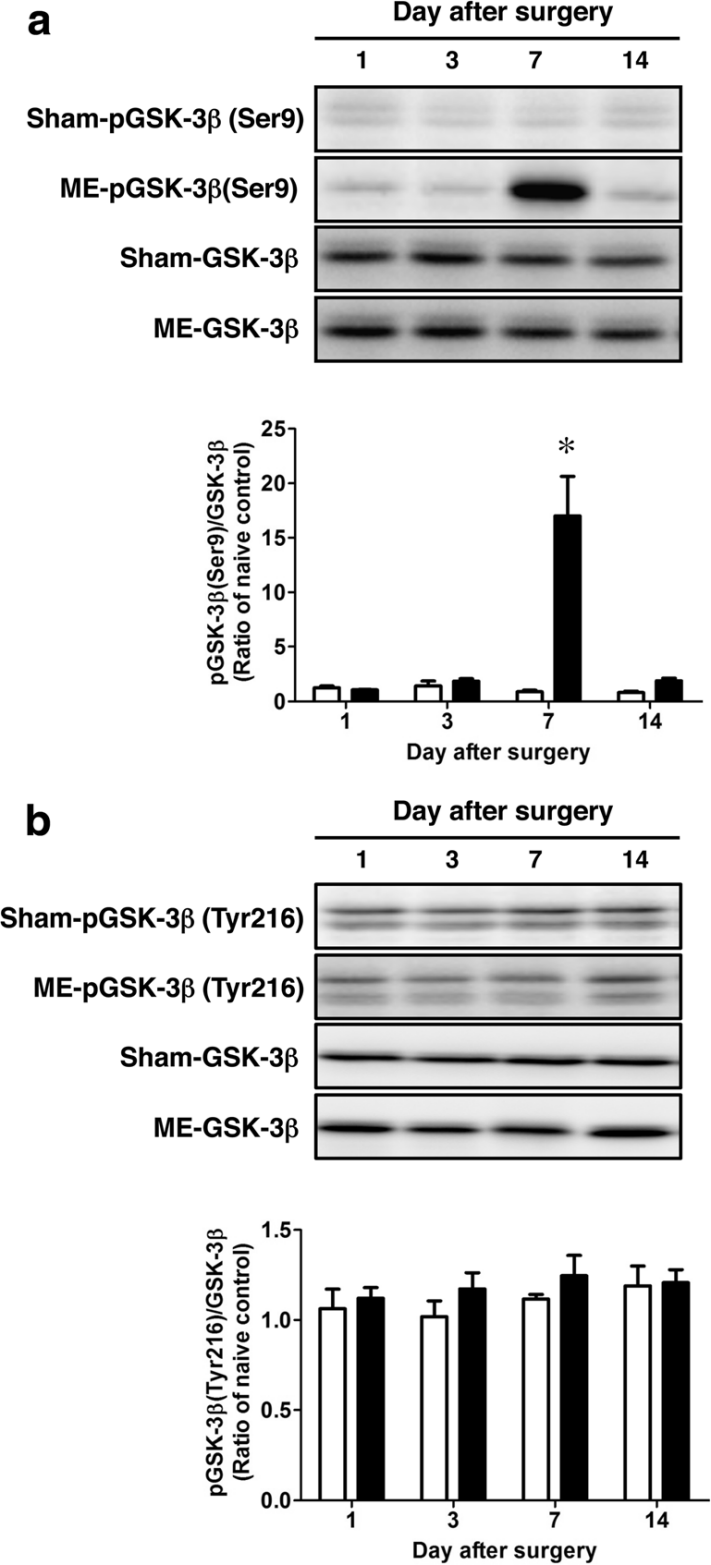
Time course of changes in the levels of phosphorylated GSK-3β after cerebral ischemia. **a** Time course of changes in the phosphorylation of GSK-3β at its Ser9 and total levels of GSK-3β in the sham- (sham; *white bars*) and ME-operated (ME; *black bars*) on days 1, 3, 7, and 14 after surgery. Bands corresponding to phosphorylated GSK-3β at Ser9 (pGSK-3β [Ser9]) and total GSK-3β (GSK-3β) were scanned, and the scanned bands were normalized by total GSK-3β on the same blot. **b** Time course of changes in the phosphorylation of GSK-3β at its Tyr216 and total levels of GSK-3β in the sham- (sham) and ME-operated (ME) on days 1, 3, 7, and 14 after surgery. Bands corresponding to phosphorylated GSK-3β at its Ser9 (pGSK-3β (Ser9)) and total GSK-3β (GSK-3β) were scanned, and the scanned bands were normalized by total GSK-3β on the same blot. Results are expressed as the mean ratio of the ME group to the non-operated (naïve) group ± SEM (*n* = 5 each). *Significant difference from the sham-operated group (*p* < 0.05)

**Fig. 2:**
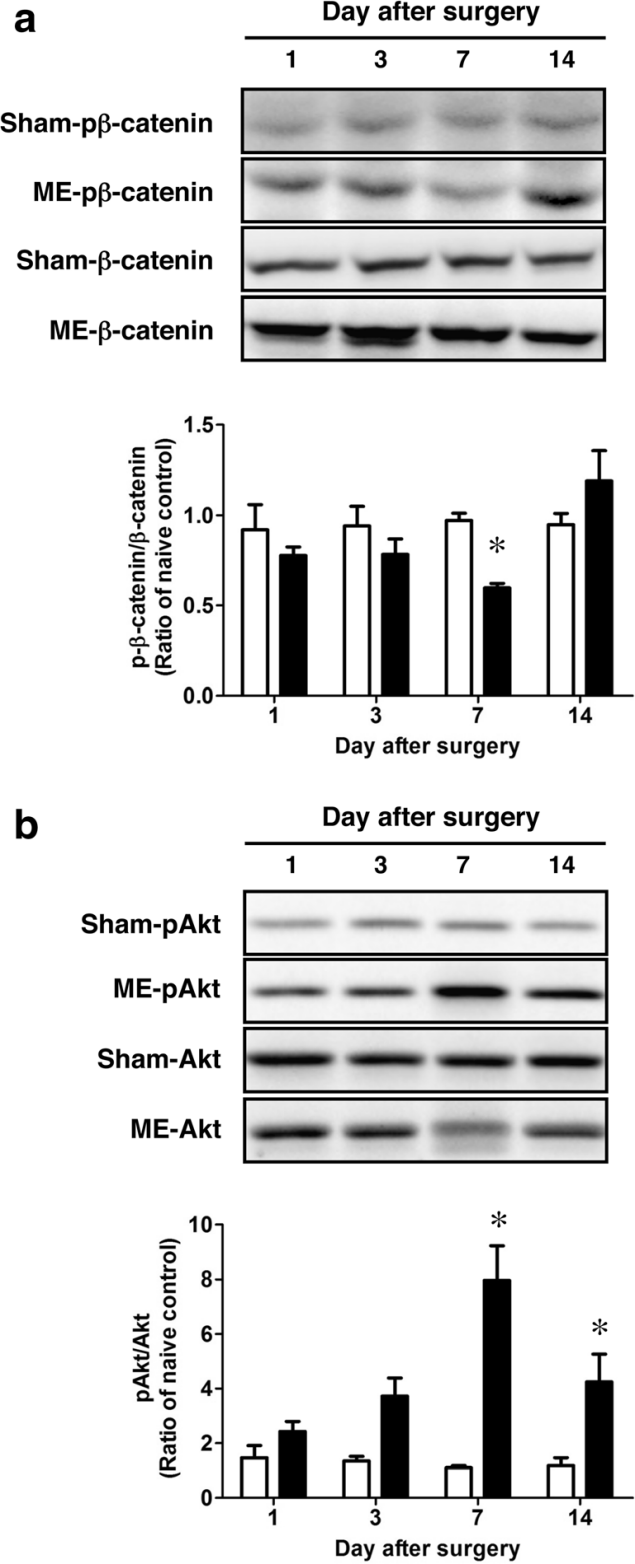
Time course of changes in the levels of phosphorylated β-catenin after cerebral ischemia. **a** Time course of changes in the phosphorylation of β-catenin and total levels of β-catenin in the sham- (sham; *white bars*) and ME-operated (ME; *black bars*) on days 1, 3, 7, and 14 after surgery. Bands corresponding to phosphorylated β-catenin (pβ-catenin) and total β-catenin were scanned, and the scanned bands were normalized by total β-catenin on the same blot. **b** Time course of changes in the phosphorylation of Akt and total levels of Akt in the sham- (sham) and ME-operated (ME) on days 1, 3, 7, and 14 after surgery. Bands corresponding to phosphorylated Akt (pAkt) and total Akt were scanned, and the scanned bands of pAkt were normalized by total Akt on the same blot. Results are expressed as the mean ratio of the ME group to the non-operated (naïve) group ± SEM (*n* = 5 each). *Significant difference from the sham-operated group (*p* < 0.05)

It is known that GSK-3β is inactivated by phosphorylation at its Ser9 and activated by that at its Tyr216. We first determined the time course of changes in phosphorylation levels of GSK-3β at Ser9 as well as at Tyr216 in the hemispheres after surgery. The phosphorylation level of GSK-3β at Ser9 of ME-operated rats was significantly increased compared with that of sham-operated rats on day 7 (Fig. 1a). In contrast, there were no significant differences in phosphorylation level of GSK-3β at Tyr216 between sham- and ME-operated rats throughout the experiment (Fig. 1b). Normally, β-catenin is phosphorylated by GSK-3β in the cytosol and is degraded by the ubiquitin-proteasome pathway. As GSK-3β was inactivated by marked phosphorylation at Ser9 on day 7 after ME, we next examined the phosphorylation level of β-catenin in the cytosolic fraction. Only on day 7 after ME, the phosphorylation level of β-catenin was decreased relative to that of sham-operated control (Fig. 2a). It is known that GSK-3β at Ser9 is phosphorylated by Akt [36]. Next, to examine the upstream of phosphorylated GSK-3β, we assessed the time course of changes in the level of phosphorylated Akt. Phosphorylation of Akt at its Ser473, which activates the enzyme, after ME gradually increased as compared with that of sham-operated rats up to day 7 (Fig. 2b). The increased phosphorylation of Akt at Ser473 remained on day 14 after ME. The greatest increase was seen on day 7 after ME (Fig. 2b).

### Cell Proliferation and Differentiation After ME

**Fig. 3:**
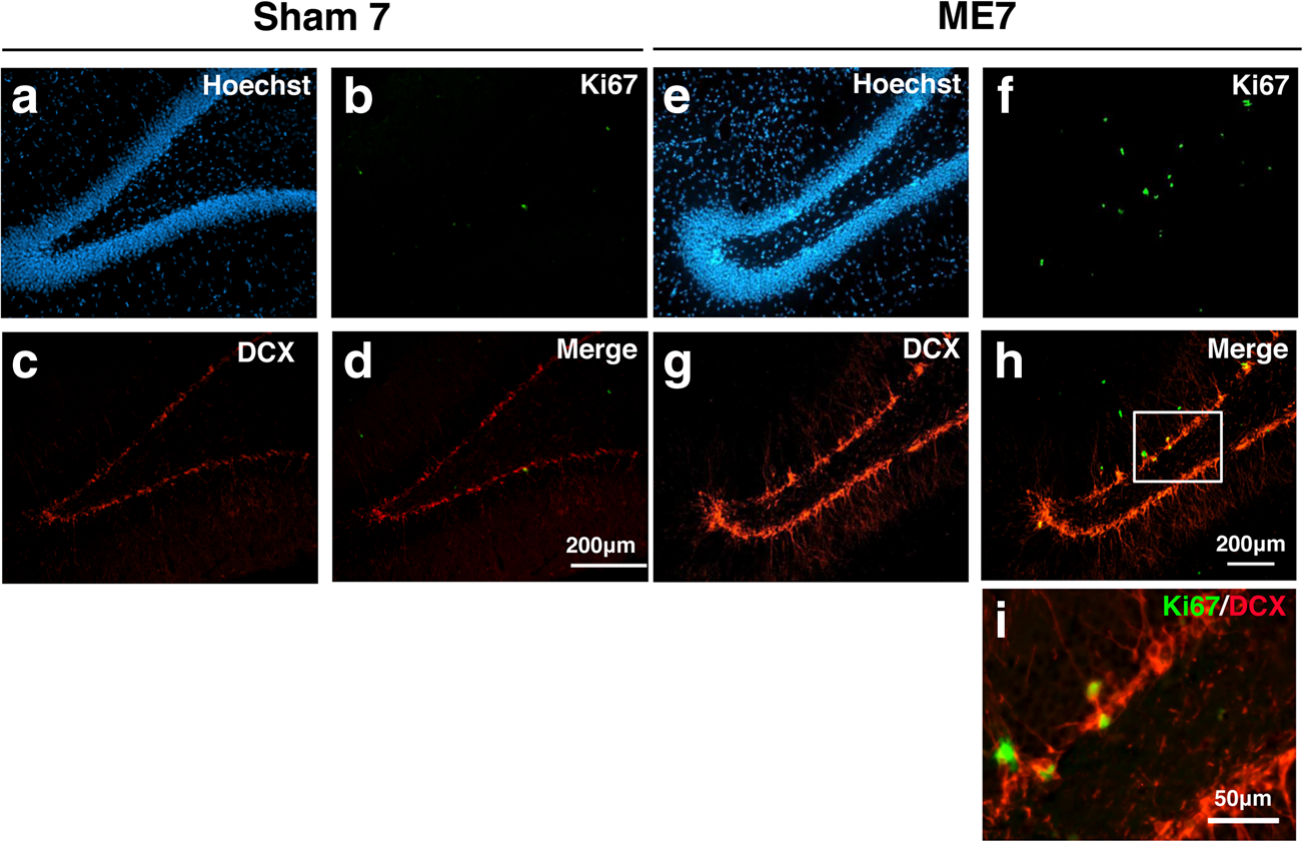
Enhancement of cell proliferation and differentiation in the hippocampal dentate gyrus on day 7 after cerebral ischemia. **a**, **b** Cells in the dentate gyrus were stained with Hoechst33342 (Hoechst). **b**, **f** Ki67-positive cells in the SGZ of the ipsilateral dentate gyrus of sham-operated (**b**) and ME (**f**) rats. **c**, **g** Ki67-positive cells (*green*) expressed a marker for immature neuron (DCX; *red*). **d**, **h** Images of double staining (merge) with Ki67 (*green*, **b**, **f**) and DCX (*red*, **c**, **g**). **i** Image of double staining with Ki67 and DCX in “H” is enlarged. *Scale bar*, 200 and 50 μm (in enlargement)

**Fig. 4:**
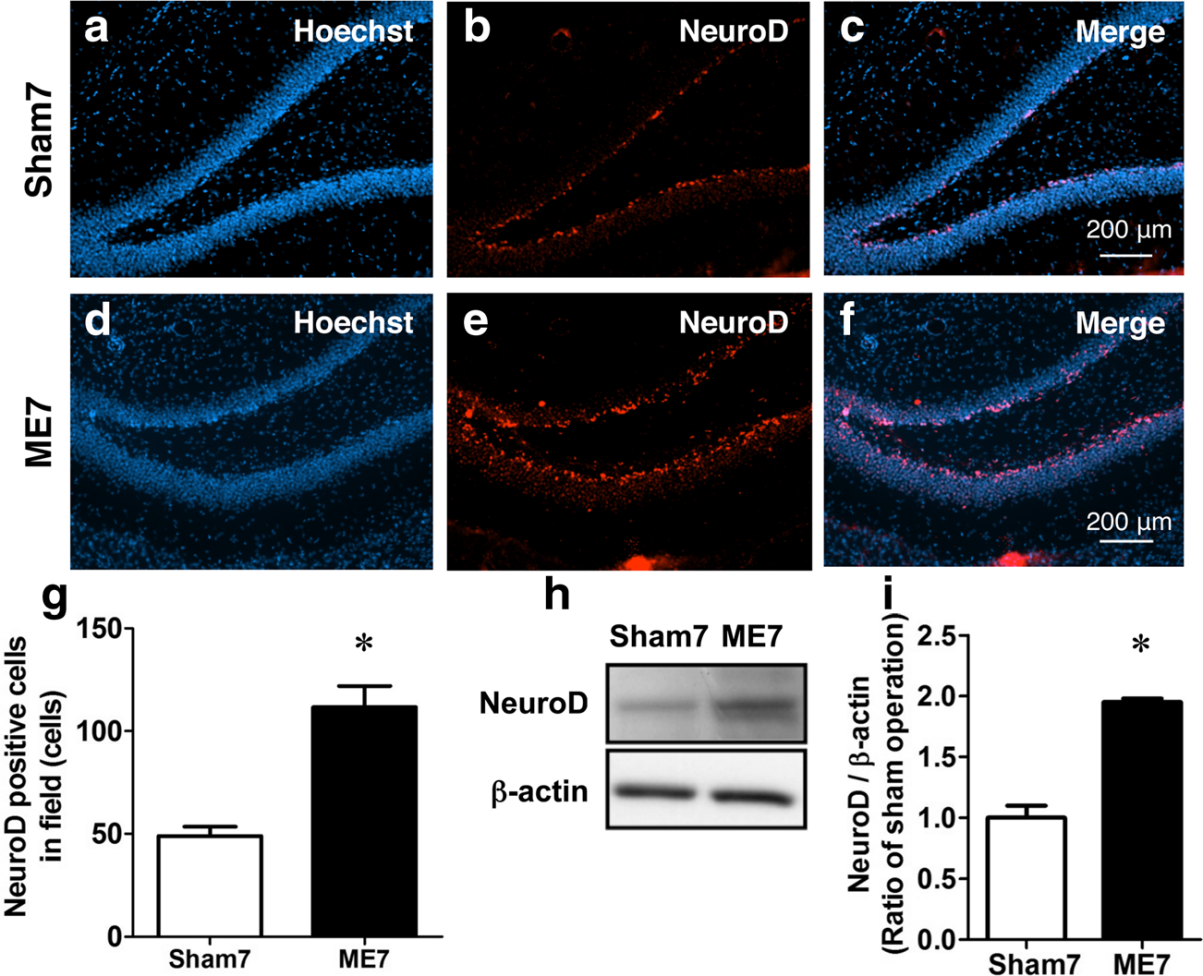
Increase in the number of NeuroD-positive cells after cerebral ischemia. Images of double staining (merge, **c**, **f**) with Hoechst 33342 (Hoechst, **a**, **d**) and for NeuroD (*red*, **b**, **e**) in the ipsilateral hippocampal dentate gyrus on day 7 after sham (**a**–**c**) and cerebral ischemia (**d**–**f**). *Scale bar*, 200 μm. **g** The number of NeuroD-positive cells in the ipsilateral SGZ of sham-operated (Sham7) and ME-operated (ME7) rats on day 7 after surgery. Values for NeuroD-positive cells are represented as the mean ± SEM. *Significant difference from the sham-operated group (*p* < 0.05). **h**, **i** Immunoblotting analysis for the level of NeuroD protein in the sham- (Sham7) and ME-operated (ME7) rats on day 7 after surgery. Bands corresponding to NeuroD and β-actin were scanned, and the scanned bands were normalized by β-actin on the same blot (**h**, **i**). Results are expressed as the mean ratio of the ME group to the sham-operated group ± SEM (*n* = 5 each). *Significant difference from the sham-operated group (*p* < 0.05)

The GSK-3β/β-catenin signaling pathway is associated with the expression of NeuroD, which plays an important role in neural differentiation. We therefore determined cell proliferation and differentiation in the hippocampal dentate gyrus on day 7 after ME at the same time period during which the phosphorylation of GSK-3β at Ser9 was significantly increased. We initially performed immunostaining using Ki67 antibody, a marker of proliferating cells, to confirm cell proliferation after ME. Ki67-positive cells were detected in the hippocampal dentate gyrus, and their number was increased in the ipsilateral dentate gyrus (Fig. 3e, f) compared with that in the sham-operated rats (Fig. 3a, b). Next, to determine whether the proliferating cells differentiated into neurons, we examined the expression of a neural marker in these Ki67-positive cells. Ki67-positive cells expressed DCX, a marker for immature neurons, on day 7 after ME (Fig. 3c–i). Furthermore, the expression of NeuroD, which plays an important role in neural differentiation, was enhanced in the ipsilateral dentate gyrus after ME (Fig. 4d–f) compared with that in the sham-operated rats (Fig. 4a–c). This increase in the number of NeuroD-positive cells was detected in the ipsilateral dentate gyrus on day 7 after ME (Fig. 4g). Immunoblotting analysis also showed that the level of NeuroD after ME was significantly increased compared with that of sham-operated rats (Fig. 4h–i).

**Fig. 5:**
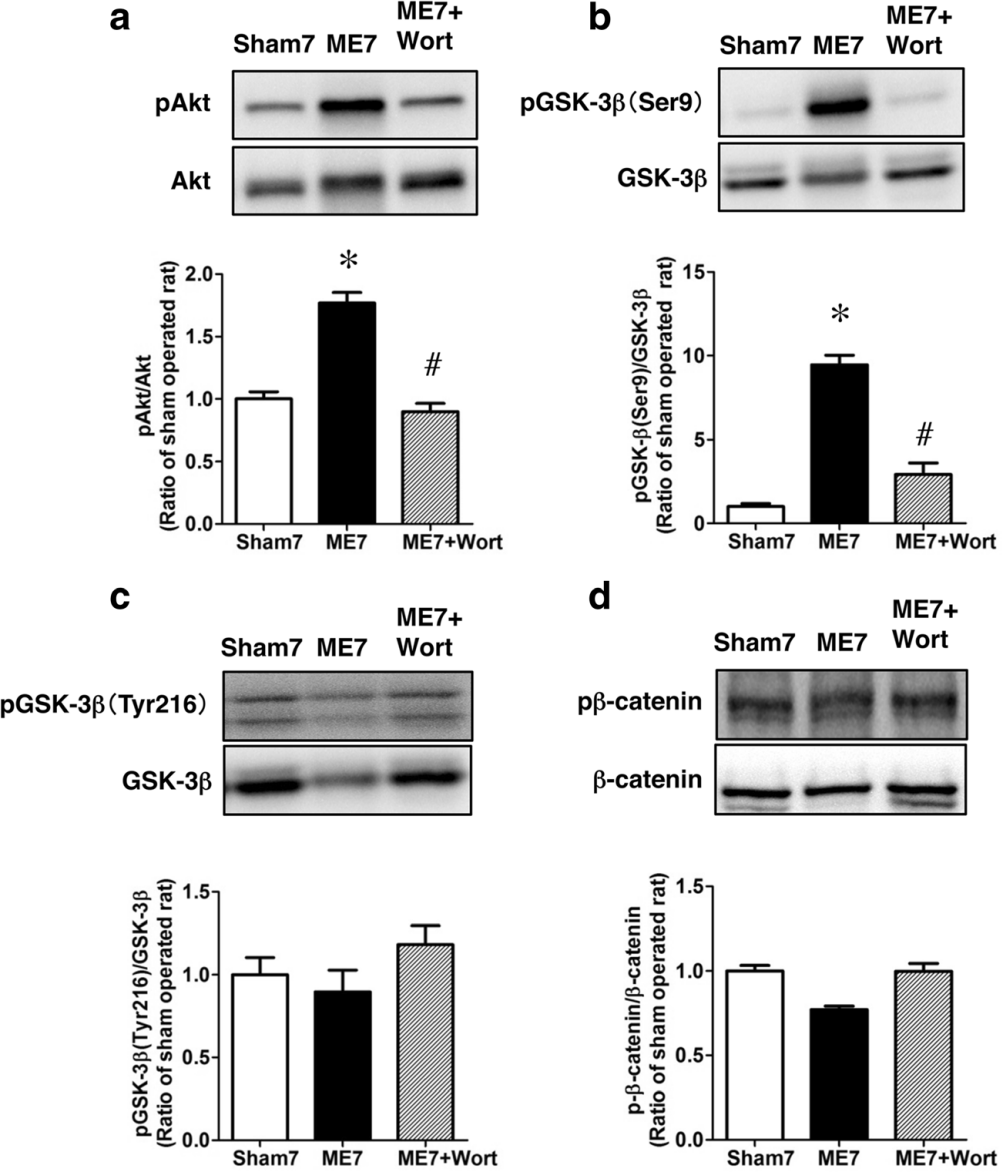
Effects of wortmannin on GSK-3β signaling. Effects of wortmannin on the phosphorylation of Akt (**a**), GSK-3β (Ser9; **b**), GSK-3β (Tyr216; **c**), and β-catenin (**d**) on day 7. Proteins from sham-operated (Sham7) and ME rats on day 7 without (ME7) or with wortmannin (ME7 + Wort) were subjected to immunoblotting with anti-phospho-Akt (pAkt), anti-phospho-GSK-3β at Ser9 (pGSK-3β [Ser9]), anti-phospho-GSK-3β at Tyr216 (pGSK-3β [Tyr216]), or anti-phospho-β-catenin (pβ-catenin) antibody. The blots were then stripped and re-probed with anti-Akt, GSK-3β, or β-catenin antibody. Bands corresponding to phosphorylated forms and non-phosphorylated forms were scanned, and the scanned bands of phosphorylated proteins were normalized by non-phosphorylated proteins on the same blot. Results are expressed as the mean ratio of the ME group to the sham-operated group ± SEM (*n* = 5 each). *Significant difference from the sham-operated group (*p* < 0.05). ^#^Significant difference from the ME-operated group (*p* < 0.05)

**Fig. 6:**
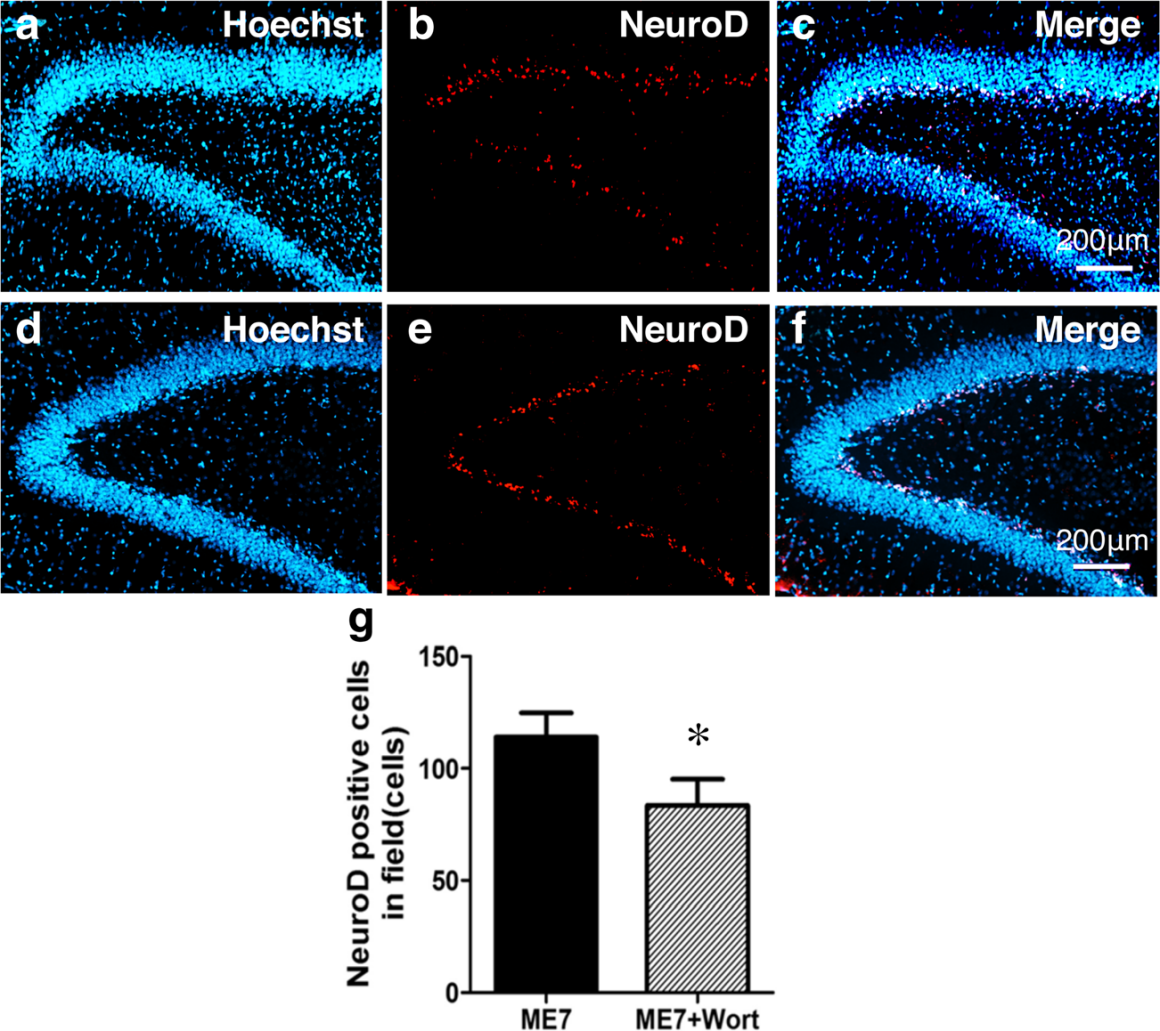
Decrease in the number of NeuroD-positive cells by treatment with wortmannin. **a**–**f** Images of double staining (merge, **c**, **f**) with Hoechst 33342 (Hoechst, **a**, **d**) and NeuroD (*red*, **b**, **e**) in the ipsilateral hippocampal dentate gyrus on day 7 after ME with wortmannin treatment (**d**, **e**, **f**). *Scale bar*, 200 μm. **g** Number of NeuroD-positive cells in the ipsilateral SGZ on day 7 after ME without (ME7) or with wortmannin (ME7 + Wort). Values for NeuroD-positive cells are presented as the mean ± SEM (*n* = 6 each). *Significant difference from the ME-operated group (*p* < 0.05)

We next assessed the effect of wortmannin, an inhibitor of PI3-K, on the phosphorylation of Akt and GSK-3β on day 7 after ME. The increased phosphorylation of Akt after ME was almost completely suppressed by the treatment with wortmannin (Fig. 5a). Also, the marked increase in phosphorylation of GSK-3β on its Ser9 after ME was attenuated by the wortmannin treatment (Fig. 5b). In contrast, phosphorylation of GSK-3β on Tyr216 was not affected by ME regardless of treatment or not with this inhibitor (Fig. 5c). Furthermore, the decreased phosphorylation of β-catenin was attenuated by treatment with wortmannin (Fig. 5d). To determine whether the Akt/GSK-3β signaling pathway contributed to neurogenesis via the expression of NeuroD, we next examined the effect of wortmannin on the number of NeuroD-positive cells in the hippocampal dentate gyrus on day 7 after ME. The number of NeuroD-positive cells in ME rats treated with wortmannin was lower than that of those in untreated ME rats (Fig. 6).

### Possibility of Involvement of IGF-1 and BDNF in GSK-3β Signaling After ME

**Fig. 7:**
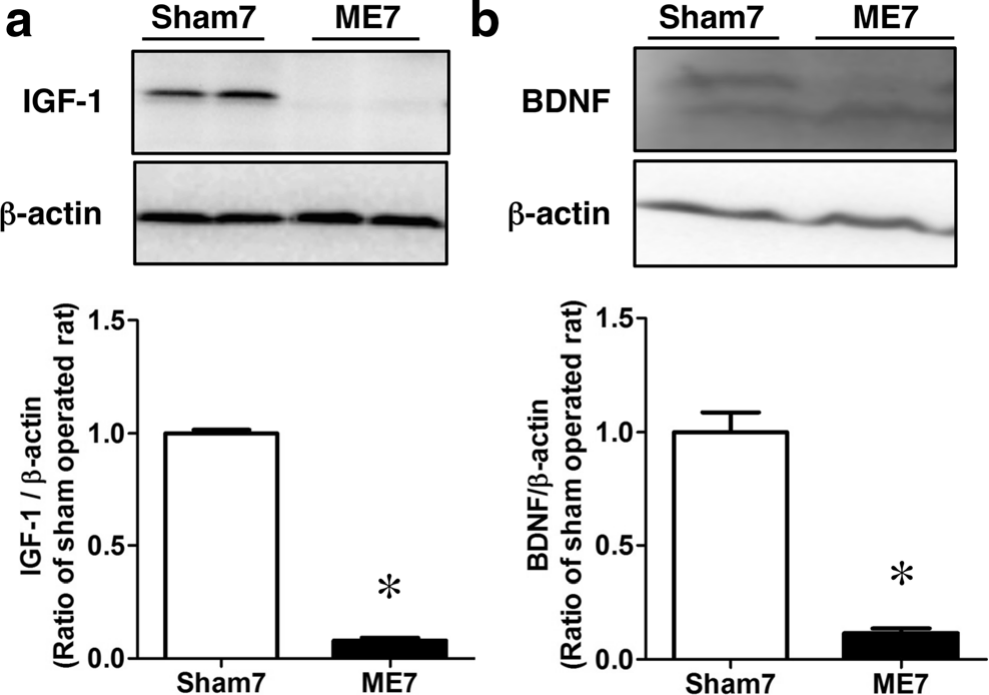
Changes in the levels of IGF-1 and BDNF after cerebral ischemia. Immunoblotting analysis for the level of IGF-1 (**a**) or BDNF (**b**) protein in the sham- (Sham7) and ME-operated (ME7) on day 7 after surgery. Bands corresponding to IGF-1 and BDNF were scanned, and the scanned bands were normalized by β-actin on the same blot. Results are expressed as the mean ratio of the ME group to the sham-operated group ± SEM (*n* = 5 each). *Significant difference from the sham-operated group (*p* < 0.05)

We next hypothesized that some growth factors are involved in the altered Akt-GSK-3β-β-catenin signaling pathway after ME. Therefore, we investigated the expression of IGF-1 and BDNF on day 7 after the operation. The expression levels of IGF-1 (Fig. 7a) and BDNF (Fig. 7b) in ME-operated rats were significantly decreased compared with those of the sham-operated rats (Fig. 7).

## Discussion

In previous studies, we demonstrated that ME increased proliferation of cells that expressed nestin, a neural progenitor marker, and DCX, a microtubule-associated protein found in migrating immature neurons. Furthermore, a few proliferated cells expressed MAP2, a neural marker, whereas GFAP was not expressed on day 28 [37]. These data imply that ME promoted cell proliferation, migration, and differentiation into neurons. However, the maximum increase in the number of proliferating cells on day 7 after ME decreased with time [37, 38]. We have also demonstrated a series of behavioral tests, including motor, sensory, reflex, and balance tests, on days 1, 7, 14, 21, and 28 after ME according to the modified neurological severity scores (mNSS) [29]. There was a significant reduction in the score of ME rats. Furthermore, we performed the sucrose preference test (SPT) and forced-swimming test (FST) as measures of post-stroke depression (PSD)-like behavior [29]. In that study, the sucrose preference of ME rats decreased at 7 days after surgery, and this decrease was sustained at least until day 28. As a decrease in sucrose consumption indicates a state of anhedonia, a core symptom of depression, we suggested that our results could be used to estimate mood disturbance of rats after ME. Furthermore, the immobility time in the FST was prolonged in ME rats [29]. These results suggested that ME-induced cerebral ischemia affected behaviors associated with depression. On the basis of our previous studies, the primary objective of the present study was to determine the intracellular signaling, including GSK pathways, in the initial stages of cerebral ischemia-induced neurogenesis for a further understanding of the pathophysiology of stroke, correlation between neurological dysfunction and ischemia-induced neurogenesis, and the development of new therapeutic targets.

GSK-3β phosphorylates many substrates and is involved in various cellular processes. Of these, β-catenin is phosphorylated by GSK-3β, leading to degradation of the former by the ubiquitin-proteasome system, whereas non-phosphorylated β-catenin in the cytosol is translocated to the nucleus, where it acts as transcriptional factor to promote the expression of NeuroD [9]. In the present study, the inactivation of GSK-3β induced by phosphorylation at its Ser9 was significantly enhanced in particular on day 7 after ME. In contrast, there were no changes in the phosphorylation of GSK-3β at Tyr216 compared with that of in the sham-operated group throughout the experiment. Changes in activity of GSK-3β depend on the region of the injury and the severity of brain ischemia models [39, 40]. Although neurogenesis is regulated by various signaling pathways, the ability of GSK-3β to play an important role in adult neurogenesis has been observed [41, 42]. For example, lithium, a GSK-3β inhibitor, increases adult neurogenesis in rodents [43]. Akt is known to phosphorylate GSK-3β at Ser 9 [36]. Thus, inactivation of GSK-3β by phosphorylation at its Ser9 after ME might be associated with Akt activation. Indeed, we demonstrated that Akt phosphorylation was enhanced on day 7 after ME. These results raise the possibility that Akt/GSK-3β/β-catenin signaling might have been enhanced after ME and contributed to neurogenesis via the expression of NeuroD.

To further determine the possible involvement of Akt-GSK-3β signaling pathways in ischemia-induced neurogenesis, we examined the effect of wortmannin, an inhibitor of the PI3-K/Akt pathway, on phosphorylation levels of Akt and GSK-3β, as well as on the expression of NeuroD. Treatment with wortmannin decreased the levels of phosphorylated of Akt and GSK-3β on its Ser9 without causing any change in phosphorylation on Tyr216 on day 7 after ME. The number of NeuroD-positive cells treated with wortmannin tended to be lower than that in the untreated control group. Our results suggest that this PI3-K-dependent Akt/GSK-3β/β-catenin signaling pathway was, at least in part, associated with the expression of NeuroD after severe cerebral ischemia. In accordance with our previous studies [37, 44], cerebral ischemia-induced cell proliferation was enhanced, and DCX, an immature neural marker, was expressed in the proliferating cells in the present study. These responses were accompanied by an increased number of NeuroD-positive cells in the ipsilateral dentate gyrus. *Neurod1*-null mice die perinatally from neonatal diabetes [45]. In addition, NeuroD-deficient neurons also die prematurely in the early stage of neurogenesis [46], suggesting that NeuroD is indispensable for neuronal differentiation and maturation at the developmental stage and for maintenance of progenitor cells. This view is consistent with the finding that conditional deletion of NeuroD1 leads to a decrease in the number of progenitors/immature neurons in the adult brain [10].

As described above, neurogenesis is regulated by various factors such as the environment, growth factors, and neurotransmitters [47–49]. Among considerable factors as important regulators of neurogenesis, it is well known that some growth factors are involved in adult neurogenesis. IGF-1 plays a crucial role in neurogenesis [50, 51]. The stimulation of cells by IGF-1 causes the inactivation of GSK-3β through a PI3-K-dependent mechanism [52]. Furthermore, the blockade of ischemia-induced GSK-3β inactivation via the inhibition of the PI3-K or of IGF-1 receptor reverses the phosphorylation of GSK-3β at its serine 9 residue [53]. Thus, IGF-1/GSK-3β signaling might be critical for ischemia-induced neuronal hyperproliferation in the hippocampal dentate gyrus region. These findings imply that the IGF-1-dependent PI3-K/Akt/GSK-3β/β-catenin signaling pathway is closely related to ischemia-induced neurogenesis. Interestingly, we demonstrated significant decreases in the level of IGF-1 on day 7 after cerebral ischemia, when the phosphorylation state of GSK-3β was at its maximum. It was also shown earlier that BDNF expression and neurogenesis in the hippocampus of depressive model animals are decreased [54, 55]. Moreover, infusion of BDNF into the hippocampus of these animals improves their depressive behavior and increases neurogenesis [56, 57]. Therefore, changes in the expression of BDNF are important as underlying determinants of depressive behavior and adult neurogenesis. We demonstrated previously that intravenous injection of NPCs on day 7 after ME improves post-stroke depression-like behaviors on day 28 after ME [29]. Also, the injection of NPCs inhibits the decrease in the level of BDNF after ME [29]. Interestingly, injected NPCs are maintained in an undifferentiated condition and might function as cells producing neurotrophic factors [29]. In the present study, the level of BDNF was rather decreased on day 7 after ME, when endogenous proliferation was at its maximum. It is well recognized that BDNF is involved in leaning and memory function, neuroprotection and/or repair, as well as in also neurite outgrowth. Although increased levels of BDNF after cerebral ischemia have been reported [58], it has been also demonstrated that the expression level of BDNF, as well as IGF, protein is significantly decreased compared with that in the appropriate control group in well-established stroke models such as the MCAO model [59–61]. Our results in the current study and previous study [29] are consistent with the latter findings. The decrease in the protein level of BDNF appears to be associated with injured brain regions, such as between ischemic penumbra and core, and the severity of ischemic models. Although cellular sources of IGF-1 and BDNF are still not clear, our results imply that the levels of IGF-1 and BDNF in NPCs per se and/or their focal concentrations around the NPCs might be critical for improving ischemic injuries. Although neurogenesis is coordinately regulated by several factors, our findings imply that an IGF-1- and/or BDNF-independent PI3-K/Akt/GSK-3β pathway was enhanced by cerebral ischemia. In this sense, it is interesting that ethosuximide, an anticonvulsant drug, induces NSC proliferation and differentiation associated with increased phosphorylation of PI3-K, Akt, and GSK-3β and decreased phosphorylation of β-catenin, suggesting that the PI3-K/Akt/GSK-3β pathway had been enhanced [62].

In conclusion, as evidence of one of the endogenous regeneration abilities after cerebral ischemia, the number of NeuroD-positive cells was increased compared with that in the sham group on day 7 after surgery. It is noteworthy that the PI3-K/Akt-dependent GSK-3β signaling pathway was involved in the expression of NeuroD, which underlies cerebral ischemia-induced neurogenesis. Furthermore, IGF-1 and BDNF might not be essential for activation of this pathway after severe cerebral ischemia. Although it remains to be determined what factors activated this pathway, the present study provides evidence that PI3-K/Akt-dependent GSK-3β signaling was involved in neurogenesis after ME. Whereas future studies will be needed to determine changes in GSK-3β phosphorylation of specific areas in the ipsilateral hemisphere, it is possible that enhancement of GSK-3β phosphorylation was not maintained due to the severe ischemic condition and that repair mechanisms after ME might not be exerted completely. As ischemia-induced neurogenesis did not repair the severe cell and tissue damages, selective and focal enhancement of GSK-3β/β-catenin pathways might be needed as therapy for ischemic injuries.
